# Reinvestigation of tris­odium dihydroxido­tetra­oxidoneptunate(VII) dihydrate

**DOI:** 10.1107/S1600536807066962

**Published:** 2007-12-21

**Authors:** Mikhail S. Grigoriev, Nikolai N. Krot

**Affiliations:** a A. N. Frumkin Institute of Physical Chemistry and Electrochemistry, Russian Academy of Sciences, 31 Leninsky prospekt, 119991 Moscow, Russian Federation

## Abstract

The title compound, Na_3_[NpO_4_(OH)_2_]·2H_2_O, contains distorted tetra­gonal–bipyramidal centrosymmetric [NpO_4_(OH)_2_]^3−^ complex anions. The Np—O distances are 1.8975 (7) and 1.8891 (7) Å in the NpO_4_ group and 2.3451 (7) Å to the OH group. Both Na atoms (one in a general position, the second in a special position on an inversion centre) have a distorted octahedral oxygen environment.

## Related literature

The structure of Na_3_[NpO_4_(OH)_2_]·2H_2_O was investigated by photographic technique with visual estimation of reflection intensities by Tomilin *et al.* (1981*a* ▶). Several other Np^VII^ compounds containing [NpO_4_(OH)_2_]^3−^ anions have been studied by photographic techniques, *viz*. Na_3_[NpO_4_(OH)_2_] (Tomilin *et al.*, 1981*b* ▶), Na_3_[NpO_4_(OH)_2_]·4H_2_O (Tomilin *et al.*, 1981*c* ▶), K_3_[NpO_4_(OH)_2_]·2H_2_O (Tomilin *et al.*, 1983 ▶). Diffractometric structure determinations have been made for [Co(NH_3_)_6_][NpO_4_(OH)_2_]·2H_2_O (Grigor’ev *et al.*, 1986 ▶), Cs_3_[NpO_4_(OH)_2_]·3H_2_O (Grigor’ev *et al.*, 1993 ▶), K_3_[NpO_4_(OH)_2_]·2H_2_O (Charushnikova *et al.*, 2007 ▶) and Na_3_[NpO_4_(OH)_2_] (Grigoriev & Krot, 2007 ▶).[Chem scheme1] 
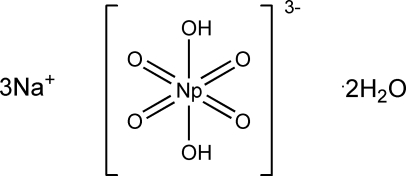


## Experimental

### 

#### Crystal data

Na_3_[NpO_4_(OH)_2_]·2H_2_O
*M* *_r_* = 440.02
Monoclinic, 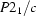

*a* = 7.8166 (3) Å
*b* = 7.7703 (2) Å
*c* = 6.8211 (2) Å
β = 112.9139 (14)°
*V* = 381.60 (2) Å^3^
*Z* = 2
Mo *K*α radiation
μ = 13.79 mm^−1^
*T* = 100 (2) K
0.12 × 0.08 × 0.02 mm

#### Data collection

Bruker Kappa APEXII area-detector diffractometer
Absorption correction: multi-scan (*SADABS*; Sheldrick, 2004 ▶) *T*_min_ = 0.522, *T*_max_ = 0.770
16264 measured reflections
2357 independent reflections
1920 reflections with *I* > 2σ(*I*)
*R*_int_ = 0.024

#### Refinement

*R*[*F* ^2^ > 2σ(*F* ^2^)] = 0.010
*wR*(*F* ^2^) = 0.021
*S* = 1.04
2357 reflections
70 parameters
3 restraints
All H-atom parameters refined
Δρ_max_ = 0.75 e Å^−3^
Δρ_min_ = −0.88 e Å^−3^

### 

Data collection: *APEX2* (Bruker, 2006 ▶); cell refinement: *SAINT-Plus* (Bruker, 1998 ▶); data reduction: *SAINT-Plus*; program(s) used to solve structure: *SHELXS97* (Sheldrick, 1997*a* ▶); program(s) used to refine structure: *SHELXL97* (Sheldrick, 1997*a* ▶); molecular graphics: *SHELXTL97* (Sheldrick, 1997*b* ▶); software used to prepare material for publication: *SHELXTL97*.

## Supplementary Material

Crystal structure: contains datablocks global, I. DOI: 10.1107/S1600536807066962/sg2217sup1.cif

Structure factors: contains datablocks I. DOI: 10.1107/S1600536807066962/sg2217Isup2.hkl

Additional supplementary materials:  crystallographic information; 3D view; checkCIF report

## Figures and Tables

**Table:** 

Np—O1	1.8975 (7)
Np—O2	1.8891 (7)
Np—O3	2.3451 (7)

**Table:** 

O1—Np—O2	91.41 (3)
O1—Np—O3	90.82 (3)
O2—Np—O3	94.67 (3)

**Table 2:** Hydrogen-bond geometry (Å, °)

*D*—H⋯*A*	*D*—H	H⋯*A*	*D*⋯*A*	*D*—H⋯*A*
O3—H3⋯O1^i^	0.815 (15)	1.976 (16)	2.7866 (10)	173 (2)
O4—H4*A*⋯O3	0.856 (17)	1.799 (17)	2.6538 (12)	178 (2)
O4—H4*B*⋯O3^ii^	0.840 (16)	1.931 (16)	2.7612 (12)	169 (2)

Symmetry codes: (i) 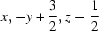; (ii) 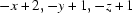.

**Fig. 1.**
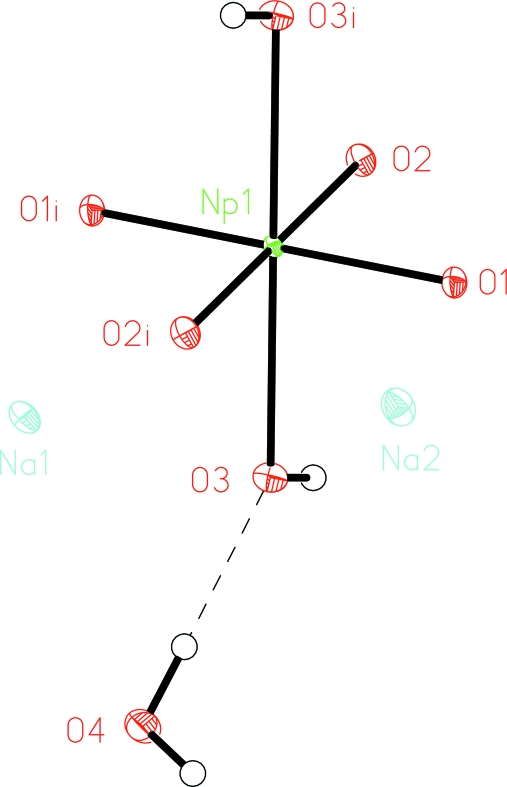
A view of (I), showing the atom-labelling scheme. Displacement ellipsoids are drawn at the 50% probability level and H atoms are represented by circles of arbitrary size. Dashed line indicates the hydrogen-bonding interaction. [Symmetry code: (i) 1 - x, 1 - y, 1 - z.]

**Fig. 2.**
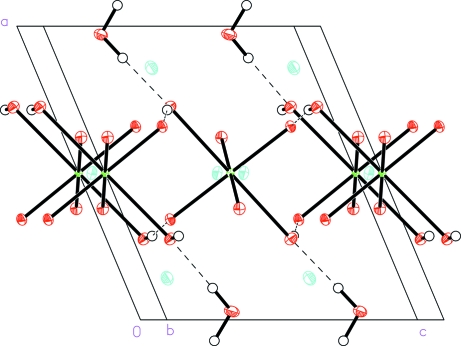
The unit cell of (I).

## supplementary crystallographic information

### Comment

The title compound, (I) (Fig. 1), contains centrosymmetric complex anions [NpO_4_(OH)_2_]^3-^ which are distorted tetragonal bipyramidal. The main bond lengths and angles in this anion are given in Table 1. The Np—O distances in the NpO_4_ group are close to the values 1.8981 (13) and 1.9012 (12) Å found in Na_3_[NpO_4_(OH)_2_] (Grigoriev & Krot, 2007). The Np—O distance to the OH group is a little longer than 2.3145 (11) Å in Na_3_[NpO_4_(OH)_2_].

Principal features of structure are the same as described by Tomilin *et al.* (1981*a*).

The Na1 atom occupies a special position on an inversion centre and has a distorted octahedral oxygen environment formed by six O atoms of two [NpO_4_(OH)_2_]^3-^ anions. The Na1 atoms and [NpO_4_(OH)_2_]^3-^ anions form columns along the [001] direction, the layers of the columns are parallel to the (100) plane (Fig. 2). The Na2 atoms and crystallization water molecules occupy general positions between the layers. The Na2 atom has a distorted octahedral oxygen environment formed by O atoms of [NpO_4_(OH)_2_]^3-^ anions and water molecules.

The OH group acts as proton donor in a hydrogen bond with an O atom of NpO_4_ group of a neighbouring anion (Table 2). This hydrogen bond is stronger than the bond formed by OH group in Na_3_[NpO_4_(OH)_2_] [the O···O distance 3.0255 (17)]. Such a difference can be one of the reasons for the elongation of the Np—O3 bond in (I). Water molecule makes two hydrogen bonds with O atoms of OH groups.

### Experimental

The starting solution for the synthesis of (I) was slightly acidic (pH ~3) 0.15 *M*^237^NpO_2_(NO_3_)_2_. The preparation of such solutions is described by Charushnikova *et al.* (2007). For the synthesis of (I), 1 ml of 0.15 *M* NpO_2_(NO_3_)_2_ aqueous solution was taken into a bubble flask, 1 ml of 5 *M* LiOH solution was added, then ozonized oxygen (10% mass of O_3_) was passed through the solution over a period of 2 h. Aliquots of 0.1 ml of the solution were put into plastic containers, 0.05, 0.1 or 0.2 ml of 16.7 *M* NaOH was added, and the containers were placed into a desiccator with granulated KOH (to absorb CO_2_ and water vapour). After four days at room temperature almost all the Np^VII^ was crystallized as bulk black crystals.

### Refinement

The H atoms of the OH-group and crystallization water molecule were located on a difference Fourier map and refined with individual displacement parameters and O—H distances restrained to 0.82 (2) and 0.85 (2) Å, respectively.

### Crystal data

**Table:**

Na_3_[NpO_4_(OH)_2_]·2H_2_O	*F*_000_ = 392
*M**_r_* = 440.02	*D*_x_ = 3.829 Mg m^−^^3^
Monoclinic, *P*2_1_/*c*	Mo *K*α radiation λ = 0.71073 Å
Hall symbol: -P 2ybc	Cell parameters from 5517 reflections
*a* = 7.8166 (3) Å	θ = 3.9–40.0º
*b* = 7.7703 (2) Å	µ = 13.79 mm^−^^1^
*c* = 6.8211 (2) Å	*T* = 100 (2) K
β = 112.9139 (14)º	Plate, black
*V* = 381.60 (2) Å^3^	0.12 × 0.08 × 0.02 mm
*Z* = 2	

### Data collection

**Table:**

Bruker Kappa APEXII area-detector diffractometer	2357 independent reflections
Radiation source: fine-focus sealed tube	1920 reflections with *I* > 2σ(*I*)
Monochromator: graphite	*R*_int_ = 0.024
*T* = 100(2) K	θ_max_ = 40.0º
ω and φ scans	θ_min_ = 3.9º
Absorption correction: multi-scan(SADABS; Sheldrick, 2004)	*h* = −14→14
*T*_min_ = 0.522, *T*_max_ = 0.770	*k* = −12→14
16264 measured reflections	*l* = −12→12

### Refinement

**Table:**

Refinement on *F*^2^	Secondary atom site location: difference Fourier map
Least-squares matrix: full	Hydrogen site location: difference Fourier map
*R*[*F*^2^ > 2σ(*F*^2^)] = 0.010	All H-atom parameters refined
*wR*(*F*^2^) = 0.021	*w* = 1/[σ^2^(*F*_o_^2^) + (0.0059*P*)^2^ + 0.087*P*] where *P* = (*F*_o_^2^ + 2*F*_c_^2^)/3
*S* = 1.04	(Δ/σ)_max_ = 0.001
2357 reflections	Δρ_max_ = 0.75 e Å^−^^3^
70 parameters	Δρ_min_ = −0.88 e Å^−^^3^
3 restraints	Extinction correction: none
Primary atom site location: structure-invariant direct methods	

### Special details

**Table:**

Geometry. All e.s.d.'s (except the e.s.d. in the dihedral angle between two l.s. planes) are estimated using the full covariance matrix. The cell e.s.d.'s are taken into account individually in the estimation of e.s.d.'s in distances, angles and torsion angles; correlations between e.s.d.'s in cell parameters are only used when they are defined by crystal symmetry. An approximate (isotropic) treatment of cell e.s.d.'s is used for estimating e.s.d.'s involving l.s. planes.
Refinement. Refinement of *F*^2^ against ALL reflections. The weighted *R*-factor *wR* and goodness of fit *S* are based on *F*^2^, conventional *R*-factors *R* are based on *F*, with *F* set to zero for negative *F*^2^. The threshold expression of *F*^2^ > σ(*F*^2^) is used only for calculating *R*-factors(gt) *etc*. and is not relevant to the choice of reflections for refinement. *R*-factors based on *F*^2^ are statistically about twice as large as those based on *F*, and *R*- factors based on ALL data will be even larger.

### Fractional atomic coordinates and isotropic or equivalent isotropic displacement parameters (Å^2^)

**Table:**

	*x*	*y*	*z*	*U*_iso_*/*U*_eq_	
Np	0.5000	0.5000	0.5000	0.00406 (1)	
Na1	0.5000	0.5000	0.0000	0.00859 (9)	
Na2	0.85815 (7)	0.32123 (6)	0.90540 (8)	0.01157 (8)	
O1	0.65824 (10)	0.56060 (10)	0.78208 (12)	0.00757 (11)	
O2	0.37890 (10)	0.71543 (9)	0.45784 (12)	0.00792 (11)	
O3	0.72436 (10)	0.59020 (9)	0.37305 (12)	0.00862 (11)	
H3	0.715 (3)	0.693 (2)	0.349 (4)	0.031 (6)*	
O4	0.97126 (12)	0.44316 (15)	0.24784 (14)	0.01657 (15)	
H4A	0.892 (4)	0.493 (2)	0.286 (5)	0.021 (6)*	
H4B	1.071 (2)	0.428 (3)	0.354 (3)	0.029 (5)*	

### Atomic displacement parameters (Å^2^)

**Table:**

	*U*^11^	*U*^22^	*U*^33^	*U*^12^	*U*^13^	*U*^23^
Np	0.00487 (2)	0.00387 (2)	0.00377 (2)	0.00001 (2)	0.00205 (1)	−0.00008 (1)
Na1	0.0114 (2)	0.0080 (2)	0.0080 (2)	0.0013 (2)	0.0056 (2)	0.0006 (2)
Na2	0.01207 (18)	0.01314 (18)	0.00847 (17)	0.00321 (15)	0.00287 (14)	−0.00015 (15)
O1	0.0084 (3)	0.0080 (3)	0.0059 (3)	−0.0006 (2)	0.0023 (2)	−0.0011 (2)
O2	0.0097 (3)	0.0055 (2)	0.0086 (3)	0.0015 (2)	0.0037 (2)	0.0006 (2)
O3	0.0091 (3)	0.0075 (3)	0.0102 (3)	0.0010 (2)	0.0049 (2)	0.0020 (2)
O4	0.0077 (3)	0.0293 (4)	0.0106 (3)	0.0028 (3)	0.0013 (3)	−0.0068 (3)

### Geometric parameters (Å, °)

**Table:**

Np—O1	1.8975 (7)	Na2—O2^i^	2.4655 (9)
Np—O2^i^	1.8891 (7)	Na2—O2^vii^	2.5150 (9)
Np—O2	1.8891 (7)	Na2—O4^viii^	2.6263 (13)
Np—O1^i^	1.8975 (7)	Na2—O4^ix^	2.7033 (12)
Np—O3	2.3451 (7)	O1—Na1^vi^	2.3223 (7)
Np—O3^i^	2.3451 (7)	O2—Na1^x^	2.3783 (7)
Na1—O1^ii^	2.3223 (7)	O2—Na2^i^	2.4655 (9)
Na1—O1^i^	2.3223 (7)	O2—Na2^xi^	2.5150 (9)
Na1—O2^iii^	2.3783 (7)	O3—H3	0.815 (15)
Na1—O2^iv^	2.3783 (7)	O4—Na2^ii^	2.3510 (10)
Na1—O3	2.5626 (8)	O4—Na2^xii^	2.6263 (13)
Na1—O3^v^	2.5626 (8)	O4—Na2^ix^	2.7033 (12)
Na2—O4^vi^	2.3510 (10)	O4—H4A	0.856 (17)
Na2—O1	2.3633 (9)	O4—H4B	0.840 (16)
			
O1—Np—O2	91.41 (3)	O2^iii^—Na1—O3	87.25 (2)
O1—Np—O3	90.82 (3)	O2^iv^—Na1—O3	92.75 (2)
O2—Np—O3	94.67 (3)	O1^ii^—Na1—O3^v^	75.46 (2)
O2^i^—Np—O2	180.0	O1^i^—Na1—O3^v^	104.54 (2)
O2^i^—Np—O1	88.59 (3)	O2^iii^—Na1—O3^v^	92.75 (2)
O2^i^—Np—O1^i^	91.41 (3)	O2^iv^—Na1—O3^v^	87.25 (2)
O2—Np—O1^i^	88.59 (3)	O3—Na1—O3^v^	180.00 (3)
O1—Np—O1^i^	180.0	O4^vi^—Na2—O1	88.70 (4)
O2^i^—Np—O3	85.33 (3)	O4^vi^—Na2—O2^i^	152.85 (4)
O1^i^—Np—O3	89.18 (3)	O1—Na2—O2^i^	66.37 (3)
O2^i^—Np—O3^i^	94.67 (3)	O4^vi^—Na2—O2^vii^	79.02 (3)
O2—Np—O3^i^	85.33 (3)	O1—Na2—O2^vii^	84.77 (3)
O1—Np—O3^i^	89.18 (3)	O2^i^—Na2—O2^vii^	87.77 (3)
O1^i^—Np—O3^i^	90.82 (3)	O4^vi^—Na2—O4^viii^	131.72 (4)
O3—Np—O3^i^	180.0	O1—Na2—O4^viii^	138.70 (3)
O1^ii^—Na1—O1^i^	180.00 (2)	O2^i^—Na2—O4^viii^	74.89 (3)
O1^ii^—Na1—O2^iii^	91.16 (3)	O2^vii^—Na2—O4^viii^	108.07 (3)
O1^i^—Na1—O2^iii^	88.84 (3)	O4^vi^—Na2—O4^ix^	93.44 (4)
O1^ii^—Na1—O2^iv^	88.84 (3)	O1—Na2—O4^ix^	71.74 (3)
O1^i^—Na1—O2^iv^	91.16 (3)	O2^i^—Na2—O4^ix^	88.88 (3)
O2^iii^—Na1—O2^iv^	180.0	O2^vii^—Na2—O4^ix^	155.55 (3)
O1^ii^—Na1—O3	104.54 (2)	O4^viii^—Na2—O4^ix^	94.370 (16)
O1^i^—Na1—O3	75.46 (2)	H4A—O4—H4B	110 (3)

Symmetry codes: (i) −*x*+1, −*y*+1, −*z*+1; (ii) *x*, *y*, *z*−1; (iii) *x*, −*y*+3/2, *z*−1/2; (iv) −*x*+1, *y*−1/2, −*z*+1/2; (v) −*x*+1, −*y*+1, −*z*; (vi) *x*, *y*, *z*+1; (vii) −*x*+1, *y*−1/2, −*z*+3/2; (viii) *x*, −*y*+1/2, *z*+1/2; (ix) −*x*+2, −*y*+1, −*z*+1; (x) −*x*+1, *y*+1/2, −*z*+1/2; (xi) −*x*+1, *y*+1/2, −*z*+3/2; (xii) *x*, −*y*+1/2, *z*−1/2.

### Hydrogen-bond geometry (Å, °)

**Table:**

*D*—H···*A*	*D*—H	H···*A*	*D*···*A*	*D*—H···*A*
O3—H3···O1^iii^	0.815 (15)	1.976 (16)	2.7866 (10)	173 (2)
O4—H4A···O3	0.856 (17)	1.799 (17)	2.6538 (12)	178 (2)
O4—H4B···O3^ix^	0.840 (16)	1.931 (16)	2.7612 (12)	169 (2)

Symmetry codes: (iii) *x*, −*y*+3/2, *z*−1/2; (ix) −*x*+2, −*y*+1, −*z*+1.
